# Autotaxin-LPA-LPP3 Axis in Energy Metabolism and Metabolic Disease

**DOI:** 10.3390/ijms22179575

**Published:** 2021-09-03

**Authors:** Anu Jose, Petra C. Kienesberger

**Affiliations:** Department of Biochemistry and Molecular Biology, Dalhousie University, Dalhousie Medicine New Brunswick, Saint John, NB E2L 4L5, Canada; Anu.Jose@dal.ca

**Keywords:** lysophosphatidic acid, autotaxin, LPA receptor, lipid phosphate phosphatase, obesity, insulin resistance, diabetes, energy metabolism, cardiomyopathy, inflammation

## Abstract

Besides serving as a structural membrane component and intermediate of the glycerolipid metabolism, lysophosphatidic acid (LPA) has a prominent role as a signaling molecule through its binding to LPA receptors at the cell surface. Extracellular LPA is primarily produced from lysophosphatidylcholine (LPC) through the activity of secreted lysophospholipase D, autotaxin (ATX). The degradation of extracellular LPA to monoacylglycerol is mediated by lipid phosphate phosphatases (LPPs) at the cell membrane. This review summarizes and interprets current literature on the role of the ATX-LPA-LPP3 axis in the regulation of energy homeostasis, insulin function, and adiposity at baseline and under conditions of obesity. We also discuss how the ATX-LPA-LPP3 axis influences obesity-related metabolic complications, including insulin resistance, fatty liver disease, and cardiomyopathy.

## 1. Introduction

The obesity epidemic presents a worldwide health crisis. The disruption in energy homeostasis during obesity predisposes to insulin resistance and type 2 diabetes mellitus (T2DM). Obesity, insulin resistance, and T2DM are linked to a multitude of comorbidities that include cardiovascular disease, non-alcoholic fatty liver disease (NAFLD), and certain cancers [1]. It is estimated that the global prevalence of insulin resistance, which is typically measured in relation to the prevalence of metabolic syndrome, is about one-quarter of the world’s population [2,3]. According to the International Diabetes Federation Diabetes Atlas, the number of adult individuals living with diabetes will increase by more than 50%, from over 450 million to over 690 million by 2045 [4]. It was also estimated that almost half of the people with diabetes are undiagnosed [4]. Diabetes drastically increases the risk of premature mortality and proves to be a major challenge for health care systems worldwide. An estimated USD 850 billion was spent on healthcare for people with diabetes globally in 2017 [4]. Cardiovascular disease is a major cause of morbidity and mortality in people with diabetes [5]. Diabetes increases the incidence of heart failure several-fold and is an independent predictor of poor outcomes, resulting in a heart failure epidemic [5]. 

**Figure 1:**
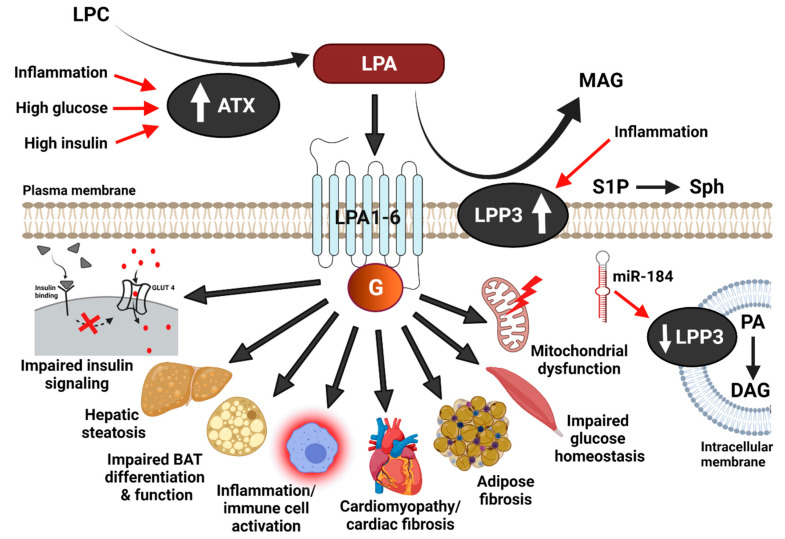
Metabolic effects of the ATX-LPA-LPP3 axis. Autotaxin (ATX) mediated hydrolysis of lysophosphatidylcholine (LPC) produces lysophosphatidic acid (LPA). LPA acts through the G-protein-coupled receptors, LPA1-6, to elicit intracellular signaling. LPP3 dephosphorylates extracellular LPA, thereby generating monoacylglycerol (MAG) and terminating LPA signaling. In addition to LPA, LPP3 can also dephosphorylate other lipids, including sphingosine-1-phosphate (S1P) to sphingosine (Sph) and intracellular phosphatidic acid (PA) to diacylglycerol (DAG). LPA signaling and ATX-LPP3 action have been implicated in metabolic complications in the context of diet-induced obesity. Mechanisms for ATX and LPP3 regulation during obesity and metabolic disease may include high insulin, high glucose, increased inflammation, and miRNA-mediated changes in gene expression. GLUT4, glucose transporter 4.

Alterations in lipid metabolism and signaling are a hallmark and contributing factor to obesity and T2DM-related morbidity [6,7,8]. When lipids accumulate in excess, particularly in non-adipose tissues, they may trigger cell stress, cellular dysfunction, and apoptotic cell death, processes that are also termed “lipotoxicity” and “lipoapoptosis” [6]. Lipids and lipid metabolites including diacylglycerols, ceramides, fatty acyl-carnitines, free fatty acids, endocannabinoids, and lipid peroxides have all been implicated in obesity and T2DM-induced metabolic complications [6]. The work published over the last decade has also revealed a strong link between lysophosphatidic acid (LPA) and metabolic disease in rodent models and humans [9]. LPA is the simplest of all glycerophospholipids, consisting of a glycerol-3-phosphate backbone and an acyl moiety [9]. LPA is not only a structural component of cellular membranes that influences membrane curvature [10] and glycolipid synthesis intermediate [8,11] but also a potent bioactive or signaling molecule [8,9]. The best-known signaling functions of LPA are mediated through its extracellular binding to G protein-coupled receptors (LPA1-6) with distinct tissue-specific expression patterns and receptor-ligand kinetics (Figure 1) [9]. LPA influences a plethora of cellular processes, including proliferation and growth, survival, development, chemotaxis, vasoregulation, and calcium dynamics [9]. 

Extracellular LPA is mainly produced from lysophosphatidylcholine (LPC) through the lysophospholipase D activity of autotaxin (ATX), which is also known as ectonucleotide pyrophosphatase/phosphodiesterase 2 (ENPP2; Figure 1) [9,12,13]. Circulating LPA levels are closely related to the ATX protein content and/or activity [9]. For example, the administration of potent ATX inhibitors diminishes plasma LPA concentrations [14,15]. Constitutive ATX deficiency in mice is embryonically lethal due to vascular and neuronal defects [12,16,17]. However, induction of ubiquitous ATX deletion in adult mice, constitutive heterozygous ATX deficiency, or fat-specific ATX deficiency does not produce any overt pathological phenotype at baseline despite a ~40–60% reduction in plasma LPA levels [12,14,16,17,18]. Similarly, long-term pharmacologic ATX inhibition is well tolerated in mice [14]. Contrary to mice with ATX reduction or inactivation, overexpression of ATX driven by the human α1-antitrypsin promoter resulted in elevated plasma LPA levels [19]. ATX-overexpressing mice exhibited bleeding diathesis and attenuated thrombosis, suggesting that the global overactivation of the ATX-LPA axis disrupts platelet function and thrombosis [19].

Extracellular LPA is generally degraded through dephosphorylation by lipid phosphate phosphatases (LPP), yielding monoacylglycerol (Figure 1) [20,21]. The mammalian LPP family consists of three enzymes—LPP1 (PPAP2A), LPP2 (PPAP2C), and LPP3 (PPAP2B)—that are encoded by distinct genes (*PLPP1/PPAP2A*, *PLPP2/PPAP2C*, and *PLPP3/PPAP2B*, respectively) [22]. In addition to LPA, LPPs catalyze the dephosphorylation of a variety of other extracellular and intracellular lipid substrates including sphingosine-1-phosphate (S1P), ceramide-1-phosphate (C1P), diacylglycerol pyrophosphate, and N-oleoylethanolamine phosphate [22]. Although in vitro catalytic activities and substrate preferences are similar for all three LPP enzymes, these proteins appear to have distinct non-redundant roles in vivo based on the targeted inactivation of the respective genes in mice [22]. LPP1 deficiency leads to increased circulating LPA levels but no overt phenotypic manifestations at baseline [23]. Similarly, LPP2 knockout mice are viable and appear normal [24]. LPA levels were not reported in these mice. LPP2 may be involved in cell cycle regulation since LPP2 knockdown in fibroblasts delayed entry into S-phase while LPP2 overexpression resulted in premature S-phase entry [25,26], effects that may be independent of changes in LPA levels [9]. In contrast to LPP1 and LPP2 knockout mice, mice with a constitutive LPP3 deficiency are not viable due to placental and extra-embryonic vascular defects [27]. Mice with liver-specific LPP3 deficiency on an apolipoprotein E knockout background have increased plasma LPA levels when fed a Western diet [28]. Adipose-specific LPP3 deficiency did not alter tissue LPA levels [29]. However, sphingolipid accumulation was reduced in adipose LPP3 knockout mice during diet-induced obesity except for S1P, which was increased in Western diet-fed mice [29]. Mice with cardiac-specific LPP3 deletion had twice as much LPA in the plasma compared to the controls [30], highlighting the contribution of cardiomyocyte LPP3 to the regulation of circulating LPA levels. In addition to being degraded by LPPs to monoacylglycerol, circulating LPA is also cleared through nonparenchymal cells in the liver via a mechanism that is largely independent of in situ degradation and involves rapid transcellular LPA uptake, contributing to the short half-life (<30 s) of circulating LPA [31]. 

In this review article, we summarize and interpret studies examining the role of ATX, LPA receptors, and LPP3 in energy homeostasis and insulin function with an emphasis on diet-induced obesity. Furthermore, we address the potential influence of ATX, LPA receptors, and LPP3 on obesity-related comorbidities, including fatty liver disease and cardiomyopathy. The role of the ATX-LPA signaling axis in adiposity and preadipocyte proliferation and differentiation was discussed elsewhere [9]. This article complements prior reviews on the role of the ATX-LPA signaling axis in obesity and impaired glucose homeostasis [9,32] while including many more recent studies and expanding the scope to discuss the involvement of LPP3 in glucose homeostasis and the ATX-LPA-LPP3 axis in metabolic cardiomyopathy. 

## 2. LPA Levels in Obesity and Metabolic Disease 

Data on LPA levels in mouse models and humans with and without obesity/T2DM remain sparse with ATX levels often being used as an indirect indicator of LPA production and concentration, as summarized in [9]. Studies in mice generally point toward an increase in circulating and/or tissue LPA levels during obesity and T2DM in rodent models and humans. For example, male mice on an FVB background showed a >60% increase in plasma LPA concentration following 13 weeks of high fat (45% kcal fat) diet feeding, which was associated with markedly increased ATX mRNA levels in perigonadal and subcutaneous white adipose tissue (WAT) [18]. Similarly, male C57Bl6/J mice fed a high fat (60% or 45% kcal fat) diet for eight or nine weeks had increased plasma LPA [33,34,35] and adipose ATX mRNA levels [33]. Female high fat-high cholesterol (42% kcal fat, 0.2% *w*/*w* cholesterol) diet-fed low-density lipoprotein receptor-null (LDLR^−/−^) mice exhibited increased unsaturated but not saturated levels of LPA in the small intestine and elevated 20:4 LPA levels in plasma 2.5 weeks post diet start [36]. However, increased plasma LPA levels in this model are likely a primary outcome of hypercholesterolemia, which is known to increase circulating LPA [37]. It should be noted that not all studies show an association between high fat diet feeding and increased LPA. WAT LPA levels were unchanged in mice fed a high fat-high cholesterol (60% kcal fat or 40% plus 0.2% *w*/*w* cholesterol) diet for eight weeks [29]. In a relatively small human cohort from Germany, plasma levels of 16:0 LPA but no other measured LPA species (18:1, 18:0, 20:4, and 22:6) were increased in individuals with obesity (BMI ≥ 30.0 kg/m^2^) compared to those with normal BMI (18.5–24.99 kg/m^2^) [38]. In 100 healthy volunteers from Poland, plasma LPA levels positively correlated with BMI [39]. Moreover, plasma LPA concentrations were higher in individuals who were overweight (BMI 25–29.99) or obese compared to those with normal or underweight BMI (<25 kg/m^2^) [39]. To avoid overestimation of blood LPA levels due to the robust LPA production following clotting-induced platelet activation, blood LPA content must be determined in plasma rather than serum [39,40,41,42]. In addition, plasma is ideally collected in siliconized tubes to prevent LPA binding to the tube surface [9]. However, baseline plasma LPA concentrations can vary by orders of magnitude among different studies [43]. As a recent report suggests, some of this variability is attributed, at least to some extent, to the artificial production and/or degradation of LPA in the collected blood sample [43]. Kano et al. [43] showed that LPA metabolism in collected blood samples can be limited by keeping the whole blood at a low temperature, followed by the addition of an ATX inhibitor to plasma, thereby keeping LPA levels constant for 30 min following blood collection. Using this technique, followed by LC-MS/MS analysis, revealed that LPA levels in plasma from mice and humans range from 40 to 50 nM with little intra-sample variation, allowing for the detection of circadian rhythms in distinct murine plasma LPA species [43]. LPA concentrations in this study were generally lower than those previously reported [18,33,34,35,39,42,44]. Therefore, future studies aimed at examining the relationship between plasma/tissue LPA and obesity/metabolic disease should use optimized blood and plasma processing procedures for a more accurate and comparable LPA determination. 

In contrast to circulating LPA, its precursor lipid, LPC, appears to decrease during obesity and T2DM. An increasing number of studies using plasma lipidomic analysis point towards an inverse relationship between levels of circulating LPC/distinct LPC species and obesity/T2DM in rodent models and humans [45,46,47,48]. These data suggest that circulating LPC and LPA levels are regulated in an opposing manner in obesity and metabolic disease. To date, relatively little is known about the role of extracellular LPC in obesity and metabolic disease. LPC appears to enhance glucose-dependent insulin secretion from pancreatic beta cells [49,50] and lower blood glucose levels in diabetic mice via stimulating adipocyte glucose uptake [51].

## 3. Regulation of ATX and LPP3 in the Context of Obesity and Metabolic Disease

In agreement with the observation that high fat feeding generally increases circulating LPA levels (Section 2), most studies to date report that increased ATX expression is associated with obesity-related insulin resistance and impaired glucose homeostasis in mice and humans [9]. In overweight or obese individuals, circulating ATX levels correlated with measures of impaired glucose homeostasis and insulin function, such as glucose infusion rate and homeostatic model assessment of insulin resistance (HOMA-IR) [52,53,54]. ATX mRNA levels in intra-abdominal adipose tissue were also higher in obese women with impaired glucose tolerance or diabetes than women with normal glucose tolerance [55]. Most, but not all, studies using mice with high fat diet-induced obesity demonstrate ATX upregulation, either through increased ATX expression in adipose tissue or circulating ATX protein/activity [18,33,35,55,56,57,58,59,60]. Similarly, ATX mRNA levels were increased in adipose tissue from obese-diabetic *db/db* mice but not gold-thioglucose-treated mice [55].

Several mechanisms were proposed to be involved in ATX upregulation in the context of diet-induced obesity and metabolic disease, including inflammation, high glucose, and insulin (Figure 1). For example, the interleukin-6 (IL-6) family of cytokines, which regulates inflammation and can induce insulin resistance, enhances ATX expression in adipocytes via glycoprotein 130 (gp130) signaling [33]. Gp130 signals through the Janus kinase (JAK)-signal transducer and activator of transcription 3 (STAT3) axis to promote ATX expression in adipocytes [33]. Oral administration of the gp130 inhibitor, SC144, in high fat-fed obese-insulin resistant mice reduced ATX expression in the adipose tissue and the plasma ATX content to levels observed in the chow-fed controls [33]. Therefore, this study suggests that gp130 is required for a robust ATX expression in adipocytes and upregulation of ATX in adipose tissue during obesity-insulin resistance [33]. Tumor necrosis factor α (TNFα) is another pro-inflammatory cytokine with the ability to impair insulin sensitivity that increases ATX expression and secretion in cultured 3T3F442A adipocytes and human adipose tissue explants [9,55]. 

In addition to being upregulated by pro-inflammatory signaling, ATX expression has also been reported to be modulated by insulin in adipocytes [56,61]. Secreted ATX activity was increased by insulin in a concentration-dependent manner in 3T3-L1 adipocytes up to 24 h, an effect that was PI3Kinase-dependent but mTORC1-independent [56]. Upregulation of ATX mRNA levels and ATX protein secretion by a 24 h and 48 h insulin treatment, respectively, was also observed in human adipose tissue explants [56]. While the treatment with the insulin sensitizer, rosiglitazone, reduced ATX mRNA in human adipose tissue and 3T3F442A adipocytes at low concentration (1 µM), it substantially increased ATX mRNA levels at high concentration (25 µM) [55,56]. The anti-inflammatory glucocorticoid, dexamethasone, inhibited insulin and rosiglitazone-induced upregulation of ATX mRNA levels in cultured human adipose tissue [61]. Moreover, mice treated with dexamethasone had lower plasma and adipose ATX activity and reduced plasma LPA and S1P levels, suggesting that the anti-inflammatory effects of dexamethasone are at least in part mediated through the ATX-LPA signaling axis [61]. Besides inflammation and insulin, glucose has been shown to increase ATX mRNA, protein, and activity in 3T3-L1 adipocytes in a time- and concentration-dependent manner [56]. Glucose also acutely upregulated ATX secretion in murine subcutaneous adipose tissue explants [56]. Taken together, inflammatory mediators, hyperinsulinemia, and elevated glucose levels during obesity-induced insulin resistance likely intersect to synergistically upregulate ATX expression and secretion from adipose tissue in a feed-forward mechanism, thereby increasing circulating ATX and LPA levels, which may further enhance inflammation and insulin resistance (Figure 1). ATX is also regulated at the post-transcriptional level, for example, by the RNA-binding proteins HuR and AUF1 [62]. However, it remains to be determined whether these post-transcriptional mechanisms also modulate ATX levels during obesity and insulin resistance.

While changes in ATX during obesity and insulin resistance are relatively well-studied, data on the regulation of LPA receptors and LPPs during metabolic disease remain scarce. Inflammatory stimuli are known to substantially upregulate LPP3 expression in various cell types [63], although this has not been tested in the context of obesity-insulin resistance (Figure 1). Mao et al. [63] showed that LPP3 upregulation during inflammation involves the NF-κB transcription factor complex. The same study also suggested that LPA itself increases LPP3 expression by activating the PI3K–AKT pathway through LPA receptors (LPA1/3), which subsequently stimulates NF-κB activity through the canonical pathway [63]. A recent study also showed that LPP3 protein levels are increased in the heart from high fat-fed mice via a mechanism that involves the microRNA (miRNA), miR-184 (Figure 1) [64]. MiRNAs are short endogenous, non-coding RNAs [64,65] that generally interact with the 3′ untranslated region of target mRNAs, thereby inducing mRNA degradation and hindering translation [66]. Nevertheless, miRNAs can also interact with other regions, including the 5′ untranslated region, coding sequence, and promoter regions [66]. Moreover, it is now understood that miRNAs can also activate translation or regulate transcription under specific circumstances [66]. MiR-184 was shown to bind to the 3′-UTR of LPP3, thereby reducing LPP3 expression in H9C2 cardiomyocytes [64]. Decreased miR-184 levels in the heart from high fat-fed mice corresponded with increased LPP3 protein levels [64]. This scenario was recapitulated in H9C2 cardiomyocytes with palmitate-induced insulin resistance, where LPP3 and miR-184 levels were increased and decreased, respectively [64]. This study further suggested that miR-184 alleviates insulin resistance in cardiomyocytes by reducing LPP3 and LPP3-mediated DAG formation from PA [64]. To date, several miRNAs have also been linked to the modulation of ATX expression [65]. These include the miR-29 family members, miR-29a-3p, miR-29b-3p, and miR-29c-3p, which were predicted by TargetScan 7.1 as miRNAs with a sequence that matches with the seed region of ATX [65,67]. MiRNAs in the miR-29 family have been implicated in the regulation of glucose homeostasis in obesity, insulin resistance, and diabetes [68,69,70]. However, it remains to be explored whether modulation of ATX expression is involved in the physiologic and metabolic actions of these miRNAs. A TargetScan screen followed by testing in cancer cells also revealed that miR-101-3p inhibits ATX expression by directly targeting a conserved sequence in the ATX mRNA 3′UTR [71]. It is possible that miR-101-3p mediated ATX regulation also contributes to altered ATX levels during obesity and insulin resistance.

## 4. Role of ATX, LPP3, and LPA Receptors in Energy Homeostasis and Obesity-Induced Metabolic Complications

### 4.1. Studies Using ATX, LPP3, and LPA Receptor Mutant Mice

Studies using ATX mutant mice have helped elucidate the role of ATX-LPA signaling in energy metabolism, obesity, and metabolic disease. High fat diet-fed (60% kcal fat, 10 weeks) mice with constitutive heterozygous ATX deficiency (ATX^+/−^) and ~50% reduced circulating LPA levels gained less body weight and had smaller fat pad weights compared to wild-type mice, which was associated with decreased adipocyte numbers in epididymal fat pads from high fat-fed ATX^+/−^ mice [59] (Table 1). Food intake and lipid accumulation in skeletal muscle, liver, and heart were similar between genotypes [59]. The reduction in diet-induced adiposity was accompanied by improved glucose and insulin tolerance in ATX^+/−^ mice [59]. This improved metabolic phenotype in ATX^+/−^ mice was also recapitulated in obese-diabetic *db/db* mice with ATX haploinsufficiency [59]. Similarly, in a more recent study, male ATX^+/−^ mice showed reduced body and fat pad weight gain and hepatic steatosis but unchanged muscle lipid accumulation and food intake during a 20-week high fat diet (45% kcal fat, 17% kcal sucrose) feeding regimen [57] (Table 1). In this study, ATX^+/−^ mice were also resistant to high fat diet-induced glucose and insulin intolerance, which was at least in part attributed to improved insulin signaling in white adipose tissue, liver, and cardiac and skeletal muscle as well as preserved insulin-stimulated muscle glucose transport [57]. Ameliorated hepatic insulin signaling in high fat-fed ATX^+/−^ mice agrees with a prior study showing that LPA can directly impair insulin signaling in primary rat hepatocytes via the LPA3 receptor [38]. Improved glucose homeostasis and insulin function in high fat-fed ATX^+/−^ mice was also associated with increased mitochondrial glucose oxidation but not fat oxidation in skeletal muscle and protection from cardiomyocyte contractile dysfunction [57]. Beneficial changes in muscle energy metabolism of high fat-fed ATX^+/−^ mice are believed to be at least in part due to the direct effect of LPA on muscles since LPA incubation of C2C12 myotubes impaired insulin signaling and mitochondrial respiration [57]. Mice with homozygous postnatal ATX inactivation and ~50% reduction in plasma LPA levels displayed an unchanged body weight and body composition but had reduced adipocyte size and inflammation on high fat (60% kcal fat) diet compared to control [72] (Table 1). Strikingly, high fat-fed mice with postnatal ATX inactivation were also protected from hepatic steatosis and inflammation and exhibited markedly improved glucose tolerance and insulin sensitivity [72]. Taken together, while constitutive heterozygous or homozygous postnatal ATX deficiency does not appear to alter energy homeostasis at baseline, it reduces adiposity/adipocyte size and improves glucose homeostasis and insulin function during obesity.

In mice with overexpression of human ATX (ENPP2), driven by the α1-antitrypsin promoter, which leads to target protein overexpression in a variety of tissues, including liver, lung, WAT, and brown adipose tissue (BAT), circulating ATX and LPA levels were moderately (~2-fold) increased [19,73]. ATX overexpression resulted in decreased BAT-related genes, for example, *Ucp1*, *Prmd16*, *Cidea*, and *Pgc1a*, in subcutaneous WAT, suggesting that increased circulating ATX-LPA levels reduce differentiation of inducible BAT in subcutaneous WAT, mirroring effects observed in cultured primary BAT preadipocytes [73]. Body weight and fat composition were similar between young-adult ATX-overexpressing and control mice on a low fat diet [73]. When fed a high fat (45% kcal fat) diet, the expression of BAT-specific genes was drastically reduced in subcutaneous WAT from ATX-overexpressing mice [73]. Moreover, periaortic BAT was enlarged with increased lipid accumulation in high fat-fed ATX-overexpressing mice [73]. Body weight gain and fat accumulation were also increased in high fat-fed ATX-overexpressing mice [73] (Table 1). Surprisingly, neither cold sensitivity, plasma inflammatory markers, nor glucose tolerance were altered in high fat-fed ATX-overexpressing compared to wild-type mice [73]. These data suggest that the ATX-LPA axis inhibits brown fat adipogenesis and, thereby, enhances susceptibility to high fat diet-induced obesity [73]. 

While studies using mice with global ATX inactivation and overexpression show that the ATX-LPA pathway negatively influences energy homeostasis and enhances high fat-diet-induced fat accumulation or adipocyte hypertrophy [57,59,72,73], studies in mice with fat-specific ATX deficiency produced more mixed results [18,59,72]. Dusaulcy et al. [18] showed that adipose-specific ATX knockout (FATX) mice, which had ~40% reduced plasma ATX levels, have more fat mass and larger adipocytes than the control upon high fat (45% kcal fat) feeding, which was associated with an upregulation of peroxisome-proliferator-activated receptor (PPAR) γ2 in subcutaneous WAT from FATX^−/−^ mice while food intake was unchanged (Table 1). These data suggest that the ATX-LPA axis reduces diet-induced obesity. Despite augmented adiposity and in agreement with studies employing global heterozygous ATX knockout mice [57,59], the glucose tolerance was increased in high fat-fed FATX^−/−^ mice [18]. In a more recent study by Nishimura et al. [59], experiments with high fat-fed (60% kcal fat) FATX^−/−^ mice essentially recapitulated those conducted using whole body ATX^+/−^ mice (Table 1). FATX^−/−^ mice showed a decreased body weight gain and were protected from the diet-induced glucose intolerance and insulin resistance compared to the control, which was associated with an improved BAT function and energy expenditure [59]. Moreover, markers of adipose tissue and systemic inflammation, including IL-6, MCP-1, and TNF-α, were reduced in high fat-fed FATX^−/−^ mice [59]. The reduced adipose tissue inflammation was also evidenced in a different high fat-fed (60% kcal fat) FATX^−/−^ mouse model [72]. Although body weight gain and fat mass were unchanged, high fat-fed FATX^−/−^ mice had smaller adipocytes compared to the control [72]. Adipose ATX deficiency also protected from the diet-induced hepatic lipid accumulation but did not change the glucose homeostasis [72]. In agreement with these data from global ATX^+/−^ and FATX^−/−^ mice, mice with an adipocyte-specific ATX overexpression, which had a five-fold increase in serum ATX and LPA levels, showed increased body and fat pad weights and enlarged adipocytes compared to the control upon high fat feeding [59] (Table 1). The same study also demonstrated that ATX promotes preadipocyte proliferation via LPA-dependent and -independent mechanisms and stimulates the immune cell activation, thus leading to an enhanced inflammation during diet-induced obesity [59]. Specifically, experiments with cultured adipocytes and immune cells showed that ATX secreted from adipocytes induces macrophage and CD8+ T-cell activation, a mechanism that likely contributes to reduced inflammation and metabolic dysfunction in high fat-fed mice with reduced ATX expression [59]. Taken together, most studies using ATX mutant mice show that the ATX-LPA axis contributes to excess adiposity and/or adipocyte enlargement and inflammation, culminating in impaired energy homeostasis during diet-induced obesity. 

Few studies have explored the role of LPP3 in obesity and associated health conditions. Federico et al. [29] showed that LPP3 is not required for the normal development of BAT or WAT in chow-fed adipocyte-specific LPP3 knockout (FLPP3^−/−^) mice. The same study also demonstrated that adipocyte-specific LPP3 deficiency reduces the accumulation of sphingolipids, specifically ceramide, sphingomyelin, sphingosine, and dihydrosphingosine, in adipose tissue, induced by a high fat (60% kcal fat) or Western diet (0.21% kcal cholesterol) [29]. Interestingly, adipose S1P levels were increased in Western diet-fed FLPP3^−/−^ mice, which was associated with augmented levels of hexadecenal, the product of S1P lyase catalyzed degradation of S1P [29]. The authors hypothesized that S1P accumulation due to LPP3 deficiency promotes irreversible degradation of S1P by S1P lyase [29]. Surprisingly, levels of PA, LPA, and DAG in adipose tissue were similar between FLPP3^−/−^ and control mice [29], suggesting that in adipocytes, LPP3 does not influence PA to DAG conversion and LPA degradation. Western diet-fed FLPP3^−/−^ mice had improved glucose tolerance and insulin sensitivity compared to the control [29] (Table 1), in agreement with the notion that the accumulation of sphingolipids, such as ceramide, can contribute to a diet-induced impairment in glucose homeostasis. On the other hand, S1P signaling has previously been implicated in the development of insulin resistance in different cell types, including hepatocytes and skeletal muscle cells [77], and circulating S1P was found to be elevated in obese mouse models and humans [78]. Thus, it remains to be determined how precisely LPP3-mediated changes in adipose sphingolipid levels influence insulin function. 

A recent study examined the role of LPP3 in cardiac function and energy metabolism using constitutive cardiomyocyte-specific LPP3 knockout (CMLPP3^−/−^) mice [30]. CMLPP3^−/−^ mice had a diminished LPP activity in the heart and markedly increased plasma LPA levels, suggesting that LPP3 is the predominant LPP in the heart and that cardiac LPP3 plays an important role in circulating LPA degradation [30]. Isolated neonatal cardiomyocytes from CMLPP3^−/−^ mice showed markedly enhanced responses to LPA stimulation, including an increased cell surface area and ERK and Rho activation [30], demonstrating that LPP3 counteracts LPA action in cardiomyocytes. CMLPP3^−/−^ mice had enlarged hearts indicative of cardiac dilatation, which was associated with progressive heart failure and premature mortality [30] (Table 1). The functional decline in CMLPP3^−/−^ hearts was age-dependent since CMLPP3^−/−^ mice younger than three months were phenotypically unremarkable [30]. The ultrastructural analysis also showed fibrosis, the absence of glycogen, and sarcomere disarray in LPP3-deficient hearts [30]. Transmission electron micrograph analysis of the myocardium from eight-month-old CMLPP3^−/−^ mice also showed extensive mitochondrial damage as was evidenced by cristae disorganization, vacuole formation, and rupture of the double membrane with deposition of an amorphous dense body [30]. Changes in mitochondrial ultrastructure in LPP3-deficient hearts were associated with functional defects as determined by respirometry analysis [30]. Specifically, basal oxygen consumption rate, mitochondrial ATP production, maximal respiration, and spare respiratory capacity were significantly reduced in LPP3-deficient cardiomyocytes [30]. On the contrary, indicators of glycolysis were increased in LPP3 knockout cardiomyocytes [30], suggesting a switch from an oxidative to glycolytic metabolism in the absence of LPP3. Superoxide production was also increased in cardiomyocytes from CMLPP3^−/−^ mice, indicative of enhanced oxidative stress that was further exacerbated by the LPA treatment [30]. Taken together, data from mice with cardiac LPP3 deficiency show that cardiomyocyte LPP3 is critical for mitochondrial function and energy homeostasis in the heart. It is therefore conceivable that the adult-onset disruption in LPP3 function accelerates a cardiac-metabolic and functional decline in the setting of diet-induced obesity, which should be tested in future studies.

Studies in mice with global LPA receptor deficiency revealed a role for LPA1 and LPA4 in the regulation of adiposity [60,74]. LPA1^−/−^ mice exhibited higher adiposity albeit lower body weight than wild-type mice on a low fat (15% kcal fat) diet, which was associated with a two-fold increase in the plasma leptin levels but unchanged lipid, glucose, and insulin levels [74] (Table 1). Interestingly, when fed a high fat diet (45% kcal fat), LPA1^−/−^ mice resisted body and adipose weight gain, which was associated with a trend towards reduced food consumption [75]. Thus, LPA1 signaling appears to limit the adipose tissue expansion at baseline but promotes increased adiposity following high fat feeding. While LPA4 deficiency did not result in changes in adiposity or metabolic homeostasis at baseline, high fat diet (60% kcal fat)-fed LPA4^−/−^ mice showed a metabolically healthy obese phenotype with an enhanced WAT expansion, which was associated with PPARγ activation and protection from adipose inflammation, hepatic steatosis, and insulin resistance [60] (Table 1). LPA4^−/−^ mice also showed an upregulation of mitochondrial and adipogenesis genes and increased production of the insulin-sensitizing adipokine, adiponectin [60]. Therefore, while data on adiposity appear to be incongruous between mouse models with ATX, LPP3, and LPA receptor modulation, glucose homeostasis and insulin function appear to be generally improved with ATX/LPA receptor inactivation during diet-induced obesity. 

### 4.2. Studies Using Pharmacological LPA Receptor and ATX Modulators and In Situ ATX Silencing

Several studies have examined the effect of the LPA1/3 antagonist, specifically Ki16425, administration on energy homeostasis and glucose metabolism in mice with genetic or diet-induced obesity. Based on mRNA analysis, using murine tissues, LPA1 and LPA3 appear to be expressed to a varying extent in insulin target tissues, including WAT, liver, skeletal muscle, and heart [76,79,80]. It has also been shown that LPA receptor levels in these tissues can markedly change during obesity in mice and humans [76,79]. When obese-diabetic *db/db* mice, which display increased adipose ATX expression, were treated with Ki16425 (5 mg/kg/day) for seven weeks, fibrosis was reduced in WAT but not liver, as determined by pro-fibrotic gene expression analysis and collagen staining [76] (Table 1). Differences in the LPA receptor expression may underlie the divergent effects of the Ki16425 administration on adipose tissue and liver fibrosis [76]. Using human adipose tissue explants, it was also determined that extracellular LPA has a direct pro-fibrotic effect, which is mediated at least in part through hypoxia-inducible factor-1α (HIF1α) [76]. Alleviation of WAT fibrosis using LPA1/3 antagonist treatment was also associated with improved insulin tolerance and reduced fasting insulin levels in *db/db* mice [76]. The same group also showed that acute LPA administration worsened glucose tolerance in both chow- and high fat (45% kcal fat)-fed mice, which was prevented using Ki16425 injections, although the insulin tolerance was not significantly altered by LPA or Ki16425 injections [34]. It was concluded that LPA acutely impairs glucose disposal through inhibition of glucose-induced insulin secretion [34]. Interestingly, chronic treatment (three weeks) with Ki16425 improved both glucose and insulin tolerance in high fat-fed mice [34] (Table 1). This improvement in systemic glucose homeostasis was paralleled by increased liver glycogen content and glucose use in skeletal muscle [34]. Similarly, a more recent study showed that a one-week treatment with Ki16425 improves insulin sensitivity and glucose tolerance in high fat diet (60% kcal fat)-fed obese mice, which was associated with reduced macrophage infiltration of the adipose tissue and plasma free fatty acid levels [33]. The same study also uncovered that inhibition of ATX via the gp130 antagonist, SC144, for one week has a similar metabolic effect compared to the Ki16425 administration in high fat-fed mice [33] (Table 1). SC144 administration reduced ATX expression in the adipose tissue and ATX and LPA levels in plasma in high fat-fed obese mice by approximately half, similar to levels observed in chow-fed mice [33]. The IL-6 family of cytokines increased ATX levels in adipocytes through gp130, contributing to IL-6-induced insulin resistance and stimulation of lipolysis in adipocytes [33]. Taken together, these studies suggest that LPA1/3 inhibition improves glucose homeostasis and inflammatory and fibrotic remodeling in adipose tissue during obesity.

Studies exploring the effect of ATX inhibition on energy metabolism, obesity, and obesity-related complications remain scarce. A relatively recent study examining whether ATX inhibition influences obesity-induced cardiomyopathy showed that circulating ATX levels positively correlate with the hypertrophy markers, atrial natriuretic peptide, and brain natriuretic peptide in a cohort of chow- and high fat (60% kcal fat)-fed mice [35]. The administration of the ATX inhibitor, PF-8380, for eight weeks attenuated high fat diet-induced cardiac hypertrophy, as was evidenced from a reduced heart weight-to-tibia length ratio, cardiomyocyte size, and cardiac hypertrophy gene expression in this group compared to vehicle-treated mice [35] (Table 1). Moreover, protection from cardiac hypertrophy was also associated with an attenuation of systolic dysfunction and cardiac dilatation [35]. In primary mouse cardiomyocytes, LPA administration increased proinflammatory and prohypertrophic gene expression, suggesting that LPA directly induces cardiac inflammation and hypertrophy [35], in agreement with other work demonstrating that LPA induces hypertrophic remodeling in neonatal rat ventricular cardiomyocytes [81] and H9C2 cells, and following myocardial infarction in mice in vivo [82]. Interestingly, in humans, circulating ATX levels correlated inversely with ejection fraction and positively with the hypertrophy marker, NT-proBNP, indicating that the ATX-LPA axis may also contribute to obesity-induced cardiomyopathy in humans [35]. In agreement with a pro-hypertrophic effect of the ATX-LPA axis on cardiomyocytes, high fat-fed (60% kcal fat) obese mice with lentiviral ATX silencing in epididymal adipose tissue showed reduced cardiac hypertrophy, which was accompanied by diminished cardiac fibrosis and steatosis [58] (Table 1). ATX silencing in WAT resulted in a drastic decline in ATX levels in adipose tissue, serum, and cardiac tissue, as well as reduced circulating LPA levels [58]. These changes were not accompanied by alterations in body weight [58]. High fat diet feeding induced a time-dependent increase in cardiac ATX levels and ATX levels correlated inversely with systolic function parameters, ejection fraction, and fractional shortening [58]. In addition to protecting from high fat diet-induced cardiac hypertrophy and fibrosis, ATX silencing also preserved the mitochondrial ultrastructure in the heart and prevented a decline in cardiac citrate synthase activity, a marker of mitochondrial abundance [58]. In addition, ATX silencing prevented a decline in cardiac ATP levels, mitochondrial oxygen consumption, and complex I activity [58]. Taken together, these studies suggest that pharmacological ATX inhibition or ATX gene silencing protect against cardiac hypertrophy, fibrosis, and mitochondrial dysfunction, thus preserving systolic function during diet-induced obesity. 

## 5. Conclusions and Future Directions

Research over the past two decades has uncovered an important role for extracellular LPA signaling and the LPA-metabolizing enzymes, ATX and LPP3, in energy homeostasis, insulin function, and obesity-induced metabolic complications. Most studies in mouse models suggest that the ATX-LPA-LPA receptor axis contributes to the obesity-related impairment of glucose homeostasis, insulin resistance, inflammation, mitochondrial dysfunction, tissue fibrosis, hepatic steatosis, and cardiomyopathy. It was also reported that increased LPA signaling during obesity promotes breast cancer progression [83], suggesting that upregulation of the ATX-LPA signaling axis could promote a variety of other obesity-related comorbidities. Whether and how LPA signaling influences energy metabolism in humans remains to be determined. Additionally, the contribution of individual LPA receptors and downstream signaling pathways to the effect of LPA on energy metabolism requires further investigation. Moreover, we are just beginning to uncover the role of LPP3 in these processes, which is complicated by the fact that this enzyme has ecto- and endo-activity against a variety of lipids in addition to LPA, including PA, S1P, and ceramide-1-phosphate. Clinical trials demonstrated that ATX and LPA1 receptor antagonists are promising candidates for the treatment of fibrotic and inflammatory conditions, specifically idiopathic pulmonary fibrosis, systemic sclerosis, and cystic fibrosis [84,85]. Therefore, it is tempting to speculate that ATX and LPA receptor inhibitors could also be used to ameliorate obesity-related inflammation and metabolic complications in humans, which should be addressed in future studies.

## Figures and Tables

**Table 1:** The influence of the ATX-LPA-LPP3 signaling axis on energy metabolism and diet-induced obesity, impaired glucose homeostasis, and metabolic comorbidities based on studies involving mice with genetic or pharmacological ATX, LPA receptor, and LPP3 modulation. HF, high fat; n.d., not determined; ↑, increased effect; ↓, decreased effect; ↔, no effect.

Mouse Model	Obesity/ Adiposity	Insulin Resistance/Glucose Intolerance	Other Comorbidities, Metabolic Parameters	Ref
HF-fed global ATX^+/−^ mice	↓	↓	Lipid accrual in muscle, liver, heart ↔	[59]
HF-fed global ATX^+/−^ mice	↓	↓	Lipid accrual in muscle ↔, liver ↓, cardiomyopathy ↓, mitochondrial dysfunction ↓	[57]
HF-fed global ATX^−/−^ mice (postnatal KO)	↔ (adipocyte size ↓)	↓	Lipid accrual in liver ↓, inflammation ↓	[72]
HF-fed global ATX over-expressing mice	↑	↔	Inflammation ↔	[73]
HF-fed FATX^−/−^ mice	↑	↓	n.d.	[18]
HF-fed FATX^−/−^ mice	↓	↓	Inflammation ↓, mitochondrial dysfunction ↓	[59]
HF-fed FATX^−/−^ mice	↔ (adipocyte size ↓)	↔	Lipid accrual in liver ↓, inflammation ↓	[72]
HF-fed adipose ATX over-expressing mice	↑	n.d.	n.d.	[59]
Western diet/HF-fed FLPP3^−/−^ mice	↔	↓	n.d.	[29]
Chow-fed CMLPP3^−/−^ mice	n.d.	n.d.	Cardiomyopathy ↑, mitochondrial dysfunction ↑	[30]
LF-fed LPA1^−/−^ mice	↑	n.d.	n.d.	[74]
HF-fed LPA1^−/−^ mice	↓	n.d.	n.d.	[75]
HF-fed LPA4^−/−^ mice	↑	↓	Lipid accrual in liver ↓, inflammation ↓	[74]
*Db/db* mice with LPA1/3 inhibition (Ki16425)	↔	↓	Fibrosis in WAT ↓, liver ↔	[76]
HF-fed C57Bl6 mice wtih LPA1/3 inhibition (Ki16425)	↔	↓	Lipid accrual in muscle, liver ↔	[34]
HF-fed C57Bl6 mice with LPA1/3 inhibition (Ki16425)	n.d.	↓	Inflammation ↓ (adipose macrophage infiltration)	[33]
HF-fed C57Bl6 mice with SC144-induced ATX reduction	↔	↓	Inflammation ↓ (adipose macrophage infiltration)	[33]
HF-fed C57Bl6 mice with ATX inhibition (PF8380)	↔	n.d.	Cardiomyopathy ↓	[35]
HF-fed C57Bl6 mice with siRNA mediated ATX silencing in WAT	↔	n.d.	Lipid accrual in heart ↓, cardiac fibrosis ↓, cardiomyopathy ↓, mitochondrial dysfunction ↓	[58]
